# Comparative Study of Combination of Oral Tranexamic Acid With Modified Kligman’s Formula Versus Oral Tranexamic Acid With Azelaic Acid 15% in the Treatment of Melasma

**DOI:** 10.7759/cureus.40908

**Published:** 2023-06-24

**Authors:** Riddhima Singh, Praveen Maheshwari, Bhushan Madke, Adarshlata Singh, Sugat Jawade

**Affiliations:** 1 Dermatology, Venereology and Leprosy, Jawaharlal Nehru Medical College, Datta Meghe Institute of Higher Education and Research, Wardha, IND

**Keywords:** social anguish., oral tranexamic acid, psychological, skin disorder, melasma

## Abstract

Background

Melasma is a persistent skin condition affecting individuals of Asian, African, and Hispanic backgrounds. It causes dark patches on sun-exposed areas of the face. The exact causes are unclear, but UV light and hormonal factors play a role. Melasma significantly impacts physical appearance and quality of life, causing emotional and social distress.

Objective

The objective was to compare the efficacy of a combination of oral tranexamic acid and modified Kligman’s formula vs. oral tranexamic acid and 15% azelaic acid.

Material and methods

This two-year interventional study occurred at the Outpatient Department of Dermatology, Venereology, and Leprosy in Sawangi, Maharashtra. It included male and female patients aged 18-50 with melasma seeking treatment. Ethical approval was obtained, and data collection involved medical histories, skin examinations, and calculating the Melasma Area and Severity Index (MASI).

Results

The study found no significant association between age groups and subject distribution in Groups A and B. Significant differences were observed in MASI scores within each group over time. There was a significant difference in mean MASI scores between Group A and Group B at the eight-week mark. A burning sensation was significantly associated with the groups, while no significant association was found for erythema.

Conclusion

This study concludes that combining oral tranexamic acid with a modified Kligman's formula is more effective in treating melasma than combining oral tranexamic acid with azelaic acid 15%.

## Introduction

Melasma therapy has relied on topical bleaching chemicals and photoprotection. Chemical peels, dermabrasion, and laser treatments are also ineffective adjuvants [1]. Tranexamic acid (TA) has been studied in melasma recently. Melasma has been treated with topical, intradermal, and oral TA [2]. Antiplasmin fibrinolytic TA may block melanocyte-stimulating paracrine melanogenic substances [1]. TA is safe and effective, although clinical trials are underpowered. Large-scale, randomized, controlled studies are needed to establish a consensus on TA for melasma [3].

There are several treatment methods to treat this condition. The disease's chronicity and recurrence frustrate the patients and the treating physician. Topical agents, lasers, chemical peels, micro-needling, dermabrasion, and oral formulations are now available. They have negative effects and poor outcomes [4]. Lasers, chemical peels, and dermabrasion need skill and expensive equipment [5]. These surgeries are costly and need many clinic visits and follow-ups. This causes patient noncompliance [6]. Thus, a treatment drug that improves outcomes without substantial adverse effects is needed.

Kligman's modified melasma treatment involves triple combination therapy (fluocinolone acetonide 0.01%, hydroquinone 2%, tretinoin 0.05%) [7]. It's safe and extensively utilized these days. Steroids may cause telangiectasia and atrophic skin changes over time, making this regimen negative. This research used tranexamic acid as an adjunct to shorten treatment time [8]. Azelaic acid is an effective treatment for melasma because it works by competitively inhibiting tyrosinase and directly killing melanocytes [9].

This study compared the effectiveness of two different treatments for melasma: a combination of oral tranexamic acid with a modified Kligman's formula and a combination of oral tranexamic acid with azelaic acid 15%. It is better than 2% hydroquinone and as effective as 4% hydroquinone but without the adverse effects of hydroquinone. This study aimed to determine which of these two treatments was more effective in treating melasma.

## Materials and methods

Study design and setting

This comparative interventional study was conducted over two years, from October 2020 to August 2022. This study was conducted at the Outpatient Department of Dermatology, Venereology, and Leprosy at the Acharya Vinobha Bhave Rural Hospital (AVBRH) in Sawangi, Wardha, Maharashtra. The study included all patients, regardless of gender, between the ages of 18-50 diagnosed with melasma and seeking treatment at the Outpatient Department of Dermatology, Venereology, and Leprosy at the AVBRH.

Inclusion and exclusion

Participants eligible for inclusion in this study are male and female patients aged 18-50 years who have been diagnosed with melasma and express their willingness to participate by providing informed consent. Patients who have previously taken other medications for melasma will be considered for inclusion, provided they undergo a washout period of one month to eliminate the potential effects of those medications.

On the other hand, patients who are currently undergoing treatment for melasma, individuals with severe systemic comorbidities, pregnant and lactating women, and patients with a history of hepatic disease or coagulopathies will be excluded from participation. Additionally, patients who are currently taking anticoagulants will not be eligible for inclusion in this study.

Ethical consideration

All participants gave written informed consent after being thoroughly informed of the study's purpose and concept. Confidentiality and privacy were ensured. The study protocol was reviewed and approved by the Datta Meghe Institute of Medical Sciences (DMIMS) (DU) Institutional Ethics Committee in December 2020 with reference number DMIMS(DU)/IEC/Dec-2020-21/9316 and published in the Journal of Pharmaceutical Research International [10].

Data collection

The patients who met the inclusion and exclusion criteria and were seeking treatment for melasma at the Outpatient Department of Dermatology, Venereology, and Leprosy at the AVBRH in Sawangi, Wardha, were enrolled in the study after obtaining clearance from the Institutional Ethical Committee (IEC). All participants provided written informed consent in their native language for voluntary study participation. A comprehensive medical history was taken, and the skin was examined, including the Melasma Area and Severity Index (MASI) calculation.

Calculation of MASI score

An assessment was conducted to determine the severity of melasma in four specific areas of the face: the forehead, right cheek, left cheek, and chin. This evaluation was based on three variables: the percentage of the total affected area (A), the darkness of the melasma (D), and the uniformity of the hyperpigmentation (H). Each variable was assigned a numerical value to represent the level of involvement. The values ranged as follows: 0 for no involvement, 1 for less than 10% involvement, 2 for involvement between 10-29%, 3 for involvement between 30-49%, 4 for involvement between 50-69%, 5 for involvement between 70-89%, and 6 for involvement between 90-100%. The darkness of the melasma (D) was compared to the normal skin, while the homogeneity of the hyperpigmentation (H) was graded on a scale of 0 to 4, as indicated in Table 1 [11].

**Table 1:** Shows grade of melasma and hyperpigmentation.

Score	Melasma Grade	Hyperpigmentation Grade
0	Normal skin color without evidence of hyperpigmentation	Normal skin color without evidence of hyperpigmentation
1	Barely visible hyperpigmentation	Specks of involvement
2	Mild hyperpigmentation	Small patchy areas of involvement <1.5 cm diameter
3	Moderate hyperpigmentation	Patches of involvement >2 cm diameter
4	Severe hyperpigmentation	Uniform skin involvement without any clear areas

The MASI score is determined by calculating the combined severity grades for darkness (D) and homogeneity (H) and multiplying them by the numerical value of the affected areas (A) as well as the percentages assigned to the four facial regions (10-30%). The total MASI score is derived by adding the following components: forehead 0.3 (D+H) A, right malar 0.3 (D+H) A, left malar 0.3 (D+H) A, and chin 0.1 (D+H) A.

Upon meeting the inclusion criteria, eligible participants were invited to participate in the study and were provided with written informed consent. They were then randomly assigned to either Group A or Group B in equal proportions (using a 1:1 allocation ratio) through the utilization of a computer-generated random number table. Assigning participants to their respective groups was conducted confidentially using the sequentially numbered, opaque, sealed envelope (SNOSE) technique [11].

Group A Participants

This study arm received treatment with a combination of oral tranexamic acid (500mg) once a day and a modified Kligman's formula cream (containing fluocinolone acetonide 0.01%, tretinoin 0.05%, and hydroquinone 2%) applied once a day at night.

Group B Participants

Patients will take oral 500mg tranexamic acid once daily, apply azelaic acid 15% gel daily at night, and use broad-spectrum SPF-30 sunscreen every three hours. Regular follow-up appointments will occur after four and eight weeks, with clinical photos taken and MASI calculated. After two months of treatment, the primary outcome measure will be the reduction of MASI score and a clinical evaluation based on photos.

Statistical analysis

The statistical analysis was conducted utilizing IBM's SPSS version 20, released in 2011 and commonly referred to as the Statistical Package for Social Sciences (IBM Corp., Armonk, NY, USA). The data was inputted into an Excel spreadsheet (Microsoft, Redmond, WA, USA), and descriptive statistics were computed for quantitative variables (mean and standard deviation) and qualitative variables (frequency and proportion). The Chi-square test was employed to assess qualitative variables, while the independent sample t-test was used to compare quantitative variables between different groups over time. Repeated measures ANOVA was employed to examine the within-group comparisons of quantitative variables, followed by post-hoc Bonferroni correction. The significance level was predetermined at 5%.

## Results

Table 2 and Table 3 revealed no statistically significant association between the age groups and the distribution of subjects in Groups A and B. The chi-square value of 3.82 suggests some degree of association, but the associated p-value of 0.147 indicates that this association is insignificant at a significance level of 0.05.

**Table 2:** Distribution of the subjects based on age group

Age groups		Groups		Total
		Group A	Group B	
21 to 30 yrs	Count	6	13	19
	%	20.0%	43.3%	31.7%
31 to 40 yrs	Count	15	10	25
	%	50.0%	33.3%	41.7%
41 to 50 yrs	Count	9	7	16
	%	30.0%	23.3%	26.7%
Total	Count	30	30	60
	%	100.0%	100.0%	100.0%
Chi-square value-3.82				
p value- 0.147				

**Table 3:** Distribution of the subjects based on gender

Gender		Groups		Total
		Group A	Group B	
Females	Count	25	24	49
	%	83.3%	80.0%	81.7%
Males	Count	5	6	11
	%	16.7%	20.0%	18.3%
Total	Count	30	30	60
	%	100.0%	100.0%	100.0%
Chi-square value-0.11				
p value- 0.73				

Table 4 presents the results of a comparison of the MASI within Group A and Group B over three timepoints (baseline, four weeks, and eight weeks) using repeated measures ANOVA. The p-values associated with the repeated measures ANOVA for Group A and Group B are 0.001, indicating a statistically significant difference in MASI scores across the three timepoints within each group.

**Table 4:** Comparison of the Melasma Area and Severity Index within the group using repeated measures ANOVA

Groups	Melasma Area And Severity Index	N	Minimum	Maximum	Mean	S.D	p-value
Group A	Baseline	30	3.0	28.2	14.77	6.36	0.001
	Four weeks	30	2.4	23.7	11.65	5.52	
	Eight weeks	30	1.2	16.2	7.89	4.51	
Group B	Baseline	30	7.5	30.0	17.34	5.40	0.001
	Four weeks	30	3.9	15.6	9.48	3.44	
	Eight weeks	30	.3	9.0	4.05	1.97	

Table 5 shows the post-hoc Bonferroni comparisons of the MASI within each group between different time intervals. The table shows the mean differences and corresponding p-values for each comparison. The p-values are all 0.001, indicating statistically significant differences between the time intervals.

**Table 5:** Comparison of the Melasma Area and Severity Index within the group between time intervals using post-hoc Bonferroni

	Mean diff	p-value	Mean diff	p-value
Baseline vs four weeks	3.12	.001	7.863	.001
Baseline vs Eight weeks	6.88	.001	13.293	.001
Four weeks vs Eight weeks	3.76	.001	5.430	.001

Table 6 shows the results of an independent sample t-test comparing the mean MASI between Group A and Group B at different time intervals. The t-tests reveal a statistically significant difference in mean MASI scores between Group A and Group B at the eight-week time interval, with a mean difference of 3.84 and a p-value of 0.001. However, no significant differences are observed at baseline or four weeks. These findings suggest that the two groups may respond differently to the treatment or intervention being assessed, as indicated by the significant difference in MASI scores at eight weeks. 

**Table 6:** Comparison of the mean Melasma Area and Severity Index (in months) between the groups using independent sample t-test SD- Standard Deviation, N- Number of Participants.

Time Interval	Groups	N	Minimum	Maximum	Mean	SD	Mean diff	p-value
Baseline	Group A	30	3	28.2	14.77	6.36	-2.57	0.096
	Group B	30	7.5	30	17.34	5.4		
Four weeks	Group A	30	2.4	23.7	11.65	5.52	2.17	0.073
	Group B	30	3.9	15.6	9.48	3.44		
Eight weeks	Group A	30	1.2	16.2	7.89	4.51	3.84	0.001

Table 7 presents the distribution of subjects based on the occurrence of side effects in Group A and Group B. There was a statistically significant association between the occurrence of a burning sensation and the groups, with a chi-square value of 6.4 and a p-value of 0.011. However, no significant association is observed for erythema. These findings suggest that the two groups may differ regarding experiencing a burning sensation as a side effect but not in terms of erythema. 

**Table 7:** Distribution of the subjects based on side effects

Side Effects			Groups		Total	Chi-square value	p-value
			Group A	Group B			
None	Absent	Count	21	18	39	0.65	0.41
		%	70.0%	60.0%	65.0%		
	Present	Count	9	12	21		
		%	30.0%	40.0%	35.0%		
Burning Sensation	Absent	Count	22	29	51	6.4	0.011
		%	73.3%	96.7%	85.0%		
	Present	Count	8	1	9		
		%	26.7%	3.3%	15.0%		
Erythema	Absent	Count	27	26	53	0.16	0.68
		%	90.0%	86.7%	88.3%		

## Discussion

Melasma is a frequent hyperpigmentation disease in India. Clinicians diagnose melasma. This research identified melasma similarly and categorized lesions by MASI score. Seventy patients were recruited and randomized into two groups of two. Group A took oral 500mg tranexamic acid OD + modified Kligman's formula (fluocinolone acetonide 0.01%, tretinoin 0.05%, and hydroquinone 2%), and Group B received azelaic acid 15% gel once a day at night alone.

In this research, Group A had a mean age of 36.43 ± 6.90 and Group B 33.50 ± 7.31. The groups had no significant association in mean age (p=0.115). Nineteen patients (31.7%) were aged 21-30, with six (20%) in Group A and 13 (43.3%) in Group B. Twenty-five patients (41.7%) were aged 31-40, with 15 (50%) in Group A and 10 (33.3%) in Group B. Nine Group A patients (30%) and seven Group B patients (23.3%) were 41-50 years old. Age and melasma therapy did not correlate significantly (p=0.147). However, Goh et al. [12] in Singapore patient research found an average age of 42.3 years. Our findings matched the Achar et al. [13] 33.45-year average.

Centro facial, malar, or mandibular involvement is clinically considered. Histological and Wood's lamp investigation indicate the epidermal, dermal, or mixed type. High-intensity UV ray exposure, pregnancy, contraception, medications, hormone treatment, and genetic abnormalities cause it [14]. Patients are aesthetically damaged and psychologically distressed due to a face skin preference and therapy refractoriness. It impairs social interaction, leisure, and emotional well-being [15].

According to the melasma pattern, 26 patients (43.3%) had centro facial type (p=1), 59 patients (98.3%) had it on both cheeks (p=0.31), 22 patients (36.7%) had it on the nose (p=0.59), and 14 patients (23.3%) had it on the chin (p=0.54). Therefore, the malar type was most predominant, followed by the centro facial type. Melasma location and treatment groups did not correlate significantly. Group A patients complained for 14.13 ± 12.27 months and Group B for 16.96 ± 13.27. Complaint duration and treatment group did not correlate significantly (p=0.39).

Contrary to Quillen et al. [16], the centro facial pattern predominates. This reveals ethnicity affects clinical presentation. It often affects Fitzpatrick skin Type III-V Asian or Hispanic women [16]. Melasma treatments vary. Modified Kligman's formula, hydroquinone, azelaic acid, kojic acid, and glycolic acid are topicals. Tranexamic acid can be used orally. Chemical peels, microdermabrasion, and lasers are procedure-based [17].

Modified Kligman's mixture is a popular topical treatment. Kligman initially utilized dexamethasone 0.1%, tretinoin 0.1%, and hydroquinone 5% on a cream basis [18]. Dexamethasone has been replaced by hydrocortisone, mometasone, fluocinolone, and fluticasone, while tretinoin and hydroquinone have been weakened. Telangiectasia, hypertrichosis, atrophy, and acneiform eruptions may result from prolonged facial corticosteroid usage [7]. Tranexamic acid, used topically, intra-lesionally as microinjections, and lately orally, lightens skin [19-21].

Wu et al. [21] found that 96% of 74 women treated for melasma with 250mg tranexamic acid tablets twice daily for six months improved. 5.4% experienced stomach pain, while 8.1% suffered hypomenorrhea. No major issues arose. Women with many pigmented lesions, such as freckles and lentigines, improved only in melasma. Tranexamic acid has varied pathogenic effects in melasma. Four to eight weeks saw improvement. Twenty-one individuals (35%) experienced no side effects, nine (15%) had a burning sensation, seven (11.7%) had erythema, eight (13.3%) had pruritis, seven (11.7%) had scaling, and two (3.3%) had stomach discomfort (p=1).

Limitations of the study

The limitation of the study was no long-term follow-up to assess relapse after the study duration.

## Conclusions

This research suggests that oral tranexamic acid and modified Kligmans formula can effectively treat melasma. Patients improved quicker than our other group with azelaic acid and maintained results for longer, minimizing treatment time. A combination of oral tranexamic acid and modified Kligman’s formula was shown to provide significantly better and faster improvements in resolving hyperpigmentation. Thus, there was a higher number of excellent responders in our first group at the end of eight weeks. The adverse effects noted were mostly minimal and self-resolving in our study. Oral tranexamic acid and modified Kligmans regimen decreased melasma recurrences; however, longer-term research is required to validate this.

## References

[1] Kim HJ, Moon SH, Cho SH, Lee JD, Kim HS (2017). Efficacy and safety of tranexamic acid in melasma: a meta-analysis and systematic review. Acta Derm Venereol.

[2] Colferai MM, Miquelin GM, Steiner D (2019). Evaluation of oral tranexamic acid in the treatment of melasma. J Cosmet Dermatol.

[3] Boissy RE, Visscher M, DeLong MA (2005). DeoxyArbutin: a novel reversible tyrosinase inhibitor with effective in vivo skin lightening potency. Exp Dermatol.

[4] Cestari T, Arellano I, Hexsel D, Ortonne JP (2009). Melasma in Latin America: options for therapy and treatment algorithm. J Eur Acad Dermatol Venereol.

[5] Rivas S, Pandya AG (2013). Treatment of melasma with topical agents, peels and lasers: an evidence-based review. Am J Clin Dermatol.

[6] Figueiredo Souza L, Trancoso Souza S (2012). Single-session intense pulsed light combined with stable fixed-dose triple combination topical therapy for the treatment of refractory melasma. Dermatol Ther.

[7] Majid I (2010). Mometasone-based triple combination therapy in melasma: is it really safe?. Indian J Dermatol.

[8] Wellington K, Wagstaff AJ (2003). Tranexamic acid: a review of its use in the management of menorrhagia. Drugs.

[9] Breathnach AS (1996). Melanin hyperpigmentation of skin: melasma, topical treatment with azelaic acid, and other therapies. Cutis.

[10] Singh R, Maheshwari P (2021). Comparative study of combination of oral tranexamic acid with modified Kligman’s formula versus oral tranexamic acid with azelaic acid 15% in the treatment of melasma. J Pharm Res Int.

[11] Bhor U, Pande S (2006). Scoring systems in dermatology. Indian J Dermatol Venereol Leprol.

[12] Goh CL, Dlova CN (1999). A retrospective study on the clinical presentation and treatment outcome of melasma in a tertiary dermatological referral centre in Singapore. Singapore Med J.

[13] Achar A, Rathi SK (2011). Melasma: a clinico-epidemiological study of 312 cases. Indian J Dermatol.

[14] Grimes PE (1995). Melasma: etiologic and therapeutic considerations. Arch Dermatol.

[15] Pawaskar MD, Parikh P, Markowski T, McMichael AJ, Feldman SR, Balkrishnan R (2007). Melasma and its impact on health-related quality of life in Hispanic women. J Dermatolog Treat.

[16] Quillen EE, Bauchet M, Bigham AW (2012). OPRM1 and EGFR contribute to skin pigmentation differences between Indigenous Americans and Europeans. Hum Genet.

[17] Sheth VM, Pandya AG (2011). Melasma: a comprehensive update: part II. J Am Acad Dermatol.

[18] Kligman AM, Willis I (1975). A new formula for depigmenting human skin. Arch Dermatol.

[19] Lee JH, Park JG, Lim SH, Kim JY, Ahn KY, Kim MY, Park YM (2006). Localized intradermal microinjection of tranexamic acid for treatment of melasma in Asian patients: a preliminary clinical trial. Dermatol Surg.

[20] Kanechorn Na Ayuthaya P, Niumphradit N, Manosroi A, Nakakes A (2012). Topical 5% tranexamic acid for the treatment of melasma in Asians: a double-blind randomized controlled clinical trial. J Cosmet Laser Ther.

[21] Wu S, Shi H, Wu H, Yan S, Guo J, Sun Y, Pan L (2012). Treatment of melasma with oral administration of tranexamic acid. Aesthetic Plast Surg.

